# Sex-specific cardiometabolic multimorbidity, metabolic syndrome and left ventricular function in heart failure with preserved ejection fraction in the UK Biobank

**DOI:** 10.1186/s12933-025-02788-4

**Published:** 2025-06-04

**Authors:** Ambre Bertrand, Xin Zhou, Andrew Lewis, Thomas Monfeuga, Ramneek Gupta, Vicente Grau, Blanca Rodriguez

**Affiliations:** 1 Computational Cardiovascular Science Group, Department of Computer Science, University of Oxford, Oxford, OX1 3QD UK; 2 Division of Cardiovascular Medicine, Radcliffe Department of Medicine, University of Oxford, Oxford, OX3 9DU UK; 3 Novo Nordisk Research Centre Oxford Ltd, Roosevelt Drive, Headington, Oxford, OX3 7FZ UK; 4 Disease Intelligence Pte Ltd., 10 Anson Road 33-10C, Singapore, 079903 Singapore; 5 Department of Engineering Science, Institute of Biomedical Engineering, University of Oxford, Oxford, OX3 7DQ UK

**Keywords:** HFpEF, cardiometabolic diseases, metabolic syndrome, machine learning, phenomapping, cardiac magnetic resonance imaging, UK Biobank

## Abstract

**Background:**

Cardiometabolic disturbances play a central role in the pathogenesis of heart failure with preserved ejection fraction (HFpEF). Due to its complexity, HFpEF is a challenging condition to treat, making phenotype-specific disease management a promising approach. However, HFpEF phenotypes are heterogenous and there is a lack of detailed evidence on the different, sex-specific profiles of cardiometabolic multimorbidity and metabolic syndrome present in HFpEF.

**Methods:**

We performed a retrospective, modified cross-sectional study examining a subset of participants in the UK Biobank, an ongoing multi-centre prospective cohort study in the United Kingdom. We defined HFpEF as a record of a heart failure diagnosis using ICD-10 code I50, coupled with a left ventricular ejection fraction (LVEF) ≥ 50% derived from cardiac magnetic resonance (CMR) imaging. We examined sex-specific differences in cardiometabolic comorbidity burden and metabolic syndrome, performed latent class analysis (LCA) to identify distinct clusters of patients based on their cardiometabolic profile, and compared CMR imaging-derived parameters of left ventricular function at rest in the different clusters identified to reflect possible differences in adverse cardiac remodelling.

**Results:**

We ascertained HFpEF in 445 participants, of which 299 (67%) were men and 146 (33%) women. The median age was 70 years old (interquartile range: [66.0–74.0]). A combination of hypertension and obesity was the most prevalent cardiometabolic pattern both in men and women with HFpEF. Most men had 2–3 clinical cardiometabolic comorbidities while most women had 1–2, despite a similar metabolic syndrome profile (*p* = 0.05). LCA revealed three distinct, clinically relevant phenogroups, namely (1) a most male and multimorbid group (n = 117); (2) a group with a high prevalence of severe obesity, abnormal waist circumference and with the highest relative proportion of females (n = 116); and finally (3) a group with an apparently lower comorbidity burden aside from hypertension (n = 212). There were significant differences in clinical measurements and medication across the three phenogroups identified. Cardiac output at rest was significantly higher in group 2 vs. group 3 (males: median 5.6 L/min vs. 5.2 L/min, *p* < 0.05; females: 5.1 L/min vs. 4.4 L/min, *p* < 0.01). Absolute global longitudinal strain was significantly lower in women in group 1 vs. group 2 (−17.6% vs. −18.5%, *p* < 0.05).

**Conclusion:**

Women with cardiometabolic HFpEF had a lower comorbidity burden compared to men despite a similar metabolic syndrome profile. Based on patients’ cardiometabolic profile, we identified three distinct subgroups which differed in body shape and mass, lipid biomarker and medication profile, as well as in cardiac output at rest both in men and women. These factors may affect disease trajectory, treatment options and outcomes in those subgroups. Subject to further validation, our findings provide a refined characterisation of the cardiometabolic HFpEF phenotype, contributing towards a better understanding of the condition to enable phenotype-specific disease management.

**Graphical abstract:**

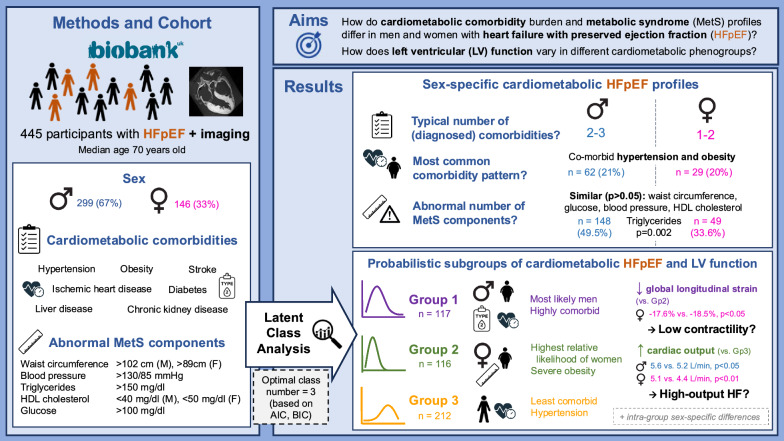

**Supplementary Information:**

The online version contains supplementary material available at 10.1186/s12933-025-02788-4.

## Introduction

Driven by chronic inflammation and metabolic disturbances, metabolic syndrome refers to a set of subclinical conditions that typically precedes overt cardiometabolic diseases such as hypertension, diabetes and ischemic heart disease [1]. These cardiometabolic conditions drive the pathogenesis of heart failure with preserved ejection fraction (HFpEF) [2, 3, 4], a condition that affects over 60 million individuals worldwide and whose prevalence keeps increasing due to older and more comorbid populations [5, 6].

Sex and age play an important role in cardiometabolic HFpEF. Compared to men, women have stiffer ventricles and arteries, smaller vasculature, and greater pulse pressure, placing them at a higher risk of developing adverse microvascular changes, diastolic dysfunction and eventually HFpEF [7, 8, 9, 10]. In addition, ageing processes differ between sexes, making the progression of cardiometabolic decline and cardiac dysfunction highly age- and sex-dependent [11, 12]. Metabolic syndrome also worsens in women during the perimenopausal stage [13, 14]. However, the limited number of sex-specific studies of HFpEF and poor representation of women in clinical trials motivate the need for further sex-specific investigations of HFpEF, especially including post-menopausal women [15, 16, 17].

Considering the multi-factorial nature of the disease, HFpEF treatment is typically adapted on an etiology-specific basis to complement first-line treatment with SGLT2 inhibitors [18, 19]. Following seminal work by Shah et al. [20], HFpEF phenotyping studies have successfully elucidated common clinical traits in different clusters of HFpEF [21, 22, 23, 24, 25, 26, 27, 28, 29, 30, 31]. However, cardiometabolic diseases and components of metabolic syndrome are not always consistently and extensively covered in these studies, and results are not always stratified by sex despite its important role in HFpEF. Cardiac function and hemodynamic parameters beyond left ventricular ejection fraction (LVEF), notably global longitudinal strain which has been associated with poorer outcomes in HFpEF [32, 33], are not always consistently accounted for. There is limited data on the variation of cardiac function across different HFpEF phenotypes including across sexes [34, 35]. The need to improve the identification and refinement of clinically distinct HFpEF phenotypes is well-acknowledged, especially phenotypes pertaining to the cardio-metabolic-renal axis, in order to improve the classification and management of HFpEF [36, 37, 38, 39].

In this study, we present a high-resolution mapping of the heterogeneities in cardiometabolic HFpEF in an elderly cohort of UK Biobank participants, ultimately aiming to help inform disease management strategies in this high-risk population. We hypothesise that cardiometabolic profiles differ in men and women with HFpEF, and that patients belonging to distinct subgroups may exhibit different extents of ventricular dysfunction. We elucidate these questions by examining the cardiometabolic comorbidity burden and metabolic syndrome in the selected cohort; extracting probabilistic phenogroups from the cohort using latent class analysis based on cardiometabolic diseases and metabolic syndrome components; and comparing whole-organ imaging-derived parameters of left ventricular function across the different cardiometabolic phenogroups identified. These analyses are stratified by sex to reflect underlying differences in men and women.

## Methods

### Study design and population

Our study sample was drawn from the UK Biobank, a prospective cohort study of over 500,000 adults living in England, Scotland and Wales, recruited between the ages of 40 and 69 years. The UK Biobank is rich in breadth and depth of data, containing information on socio-demographic and lifestyle factors, and clinical measurements recorded at multiple timepoints since recruitment. Linkage to hospital episode statistics (HES) is available for most of the cohort, while primary care records are linked for about 45% of the entire study cohort [40]. The UK Biobank imaging sub-study recalled about 10% of the original study cohort for a multi-organ imaging assessment, which included a cardiac magnetic resonance (CMR) scan performed on a 1.5 Tesla scanner (MAGNETOM Aera, Syngo Platform VD13A, Siemens Healthcare). Full details of the imaging protocol are available in [41, 42].

In this study, we performed a retrospective cohort study with hybrid prevalence/incidence analysis, with a modified cross-sectional component considering prevalent and incident cases at the time of the imaging assessment (see Cohort Definition below). We examined the relations between cardiometabolic comorbidity burden, metabolic syndrome components and left ventricular function in patients with HFpEF, stratified by sex. As a subgroup analysis, we compared the cardiometabolic profile of patients with prevalent vs. incident HFpEF at time of imaging, to support the design rationale and mitigate any selection bias that could arise from the inclusion of both in our study.

Our study is reported in line with the STROBE (Strengthening the Reporting of Observational Studies in Epidemiology) statement.

### Ethical considerations

General ethical approval was granted for UK Biobank studies by the United Kingdom's National Health Service Research Ethics Service (11/NW/0382). Participants provided written informed consent for their data to be stored and used for research purposes. This study was conducted under UK Biobank Application Number 116292.

### Cohort definition

Our study sample consists of UK Biobank participants with HFpEF, including cases of prevalent HFpEF diagnosed before the time of imaging (mean time since diagnosis: 5.7 years) and incident HFpEF diagnosed after the imaging assessment (mean follow-up time: 3.3 years). The rationale behind this hybrid approach was to capture HFpEF exhaustively considering that the age of participants in the UK Biobank is typically lower at assessment compared to the typical onset of HFpEF as evidenced by low baseline prevalence, coupled with the fact that HFpEF is commonly underdiagnosed, especially when co-existing with cardiometabolic conditions [43, 44]. To ascertain HFpEF, we first identified all UK Biobank cases with a HF diagnosis by retaining participants with a non-null date of first report of an ICD-10 code I50 in HES, primary care, death registry data and self-reported diagnoses of HF (UK Biobank field number 131354). To further classify HF based on LVEF, we retained participants with CMR image-derived LVEF values greater or equal to 50% (fields 310602242024103).

### Baseline covariates

We considered the following demographic covariates: sex (UK Biobank field 31), ethnic background (21000), age as calculated between date of birth (33) and date at which the imaging data was recorded (53), as well as the following socio-economic variables: qualifications (6138), employment status (6142) and access to private healthcare (4674). We also considered the following lifestyle and anthropomorphic covariates: body mass index (BMI) calculated using height (50) and weight (21002), waist-to-hip ratio calculated using waist circumference (48) and hip circumference (49), smoking status (20116), and alcohol consumption (1558). We included the following clinical covariates: diastolic blood pressure (DBP) (4079), systolic blood pressure (SBP) (4080), triglycerides (30870), high-density lipoprotein (HDL) cholesterol (30760), blood glucose (30740) and cholesterol-lowering medication, blood pressure medication and insulin (6153, 6177). To account for possible existing cardiac structural and functional abnormalities, we included known cardiomyopathies (131338, 131340) and history of cancer, including lung and breast cancer (20001), as detailed information on cardiotoxic treatments or chest radiotherapy was not available. For each covariate, we used the most recent measurement obtained prior to or on the date of the imaging assessment.

### Outcomes

Our study considers two key outcomes: cardiometabolic burden, whereby we consider cardiometabolic disease diagnoses and metabolic syndrome components, and CMR imaging-derived parameters of left ventricular function, in our HFpEF cohort.

#### Definition of cardiometabolic disease

We defined cardiometabolic diseases as ischemic heart disease, hypertension, diabetes, obesity, chronic kidney disease, liver disease (primarily metabolic dysfunction-associated fatty liver disease (MASLD)) and stroke. We used ICD-10 codes to identify each disease category (Table 1). We further ascertained hypertension and obesity based on clinical biomarker thresholds, as established by recent clinical guidelines, using data collected at the assessment centre. Individuals with a SBP ≥ 140 mmHg and/or DBP ≥ 90 mmHg were classified as hypertensive [45]. Those with body mass index (BMI) ≥ 30 kg/m^2^, or BMI ≥ 25 kg/m^2^ coupled with waist-to-height ratio (WtHR) ≥ 0.5, were classified as obese [46].

**Table 1:** Cardiometabolic diseases included in the study, as defined by the International Classification of Diseases (ICD)

Disease category	ICD-10 code	Description	UK Biobank field
Ischemic heart disease	I11	Hypertensive heart disease	131288
	I13	Hypertensive heart and renal disease	131292
	I20	Angina pectoris	131296
	I21	Acute myocardial infarction	131298
	I22	Subsequent myocardial infarction	131300
	I24	Other acute ischemic heart disease	131304
	I25	Chronic ischemic heart disease	131306
Hypertension	I10	Essential primary hypertension	131286
	I15	Secondary hypertension	131294
	O16	Unspecified maternal hypertension	132192
Diabetes	E10	Insulin dependent diabetes mellitus	130706
	E11	Non-insulin dependent diabetes mellitus	130708
	E14	Unspecified diabetes	130714
Obesity	E66	Obesity	130792
Chronic kidney disease	N18	Chronic renal failure	132032
Liver disease	K76	Other diseases of liver (primarily including MASLD)	131670
Stroke	I61	Intracerebral haemorrhage	131362
	I62	Other nontraumatic intracranial haemorrhage	131364
	I63	Cerebral infarction	131366
	I64	Stroke, not specified as haemorrhage or infarction	131368

Each corresponding UK Biobank field refers to the date of first report of respective ICD-10 codes. MASLD: metabolic dysfunction-associated fatty liver disease

#### Definition of metabolic syndrome

We identified abnormal metabolic syndrome components based on the following criteria: waist circumference >102 cm in men or >89 cm in women, blood pressure >130/85 mmHg, fasting triglyceride level >150 mg/dl, fasting HDL cholesterol level <40 mg/dl in men or 50 mg/dl in women, and fasting blood glucose >100 mg/dl [1]. In addition to reporting abnormalities in individual components, we reported cases of clinically-defined metabolic syndrome using the NCEP ATP III definition, defined as the presence of three or more of these criteria [1].

#### Imaging-derived parameters of left ventricular function

We included the following imaging parameters, measured at rest: LVEF (UK Biobank fields 22420, 24103, 31060), LV end-diastolic volume (22421, 24100, 31061), LV stroke volume (22423, 24102, 31064), cardiac output (22424, 24104), and LV global longitudinal strain (22181). These parameters were computed from the raw imaging data using automated software and in-house algorithms that were developed in previous studies [47, 48, 41, 42]. Details of the verification, validation, and quality control measures are available in respective publications [47, 48, 41, 42].

### Statistical analysis

All statistical analyses were performed using Python version 3.8 and R version 4.1.1.

#### Statistical tests

To compare variable distributions across different groups, normality of continuous variable distributions was assessed using the Shapiro–Wilk test. For comparisons of variables across two groups only, the independent samples t-test was used to compare normal distributions. The t-test was only performed if the sample size was ≥ 30 to preserve the validity of the Central Limit Theorem assumption. The Mann–Whitney U-test was used for non-normal distributions and holds regardless of sample size. Multiple comparison corrections were not applied here because each variable was tested independently between the two groups considered, using a single test per variable. For comparison across three groups, normally distributed variables were compared using one-way analysis of variance (ANOVA) and non-normally distributed variables were compared using the Kruksal-Wallis test. Post-hoc pairwise *p*-values were calculated using the Tukey HSD test after ANOVA, or Dunn’s test for the Kruksal-Wallis test, and further adjusted using Bonferroni correction to adjust those *p*-values by multiplying each pairwise *p*-value by the number of comparisons, i.e. 3. For the comparison of two or more categorial variable distributions, we used the Chi-Squared test. For comparison across three categorical groups using the Chi-Squared test, multiple comparison corrections were applied using the Bonferroni correction. All statistical tests were two-sided. Statistical significance was defined as *p* ≤ 0.05. Median and interquartile range (IQR) are reported for continuous variables, while count and percentage are reported for categorical variables.

#### Missing data

Missing count and percentage of the full sample are reported for all baseline variables as part of the cohort description. For the outcome analysis of imaging-derived parameters, we removed outliers with an LV end-diastolic volume (LVEDV) ≥ 500 ml and imputed values for imaging parameters that were missing completely at random, using median imputation. To mitigate bias and ensure the robustness of these results, we repeated the same outcome analysis after discarding cases that did not have a full set of imaging parameters (see Supplementary Materials).

#### Model-based clustering: latent class analysis

HFpEF subgroups were established using latent class analysis (LCA), a collection of unsupervised learning approaches that estimate unobserved groups in a population given a set of observed indicators. As opposed to k-means or hierarchical clustering methods, which make “hard” class assignments based on distances within and between data-driven clusters, LCA assumes that the data being treated comes from an underlying model which contains hidden subgroups, to which each datapoint i.e. patient is probabilistically assigned to. Thus, in this context, a model-based clustering approach is beneficial compared to heuristic clustering, as it tends to reflect reality more closely both in terms of probabilistic class belonging and the existence of an underlying distribution, therefore making results more applicable to other cohorts.

The indicator variables considered in our analysis include binary variables representing the presence or absence of individual cardiometabolic diseases as defined earlier; the presence or absence of abnormal individual metabolic syndrome components; and male or female sex. We chose to perform LCA on the full cohort with sex as an indicator variable, instead of performing two distinct LCAs in the male and female subgroups, to avoid further subgrouping into smaller clusters that may be less representative. We calculated pairwise Phi coefficients to measure the association between all possible pairs of these binary variables, to avoid including highly associated variables in the model. To select the optimal number of classes, consecutive models were fit with an increasing number of classes. The following metrics were computed: Akaike Information Criterion (AIC), Bayesian Information Criterion (BIC), and the bootstrap likelihood ratio (LR) test. AIC favours model fit and complexity, capturing more complex patterns, while BIC penalises complexity and is less prone to overfitting. The aim is to minimise both, and select a model that fits the data well while being neither too simple nor too complex. The LR test compares the fit of two models with a different number of classes by evaluating whether adding an additional class significantly improves the model fit, thus providing another possible layer of validation to AIC and BIC. LCA was implemented in R using the *poLCA* package. We ran models with a varying number of possible classes ranging between 1 and 7, with 5000 maximum iterations and 20 random starts to ensure reproducibility [49]. The LR test had 50 bootstrap replications. *poLCA* uses the expectation–maximization algorithm, which can handle missing data under the assumption that the data is missing at random and estimates the model parameters based on the available data.

#### Regression

A linear regression line was fit and the R^2^ coefficient calculated to quantify the relationship between individual metabolic syndrome components and parameters of left ventricular function.

### Subgroup analyses

To mitigate possible bias introduced by the consideration of patients with prevalent and incident HFpEF relative to the time of imaging, we carried out a subgroup analysis comparing the cardiometabolic profile of those two subgroups to identify possible differences between them, which may affect the way results are interpreted. To achieve this, we repeated the statistical tests carried out to compare cardiometabolic comorbidities, abnormal metabolic syndrome components and clinically-defined metabolic syndrome in patients with prevalent vs. incident HFpEF at the time of assessment.

## Results

### Cohort description

The study cohort consisted of 445 participants with HFpEF. The cohort was mostly male (n = 229, 67%), elderly (median age of 70 years), of white ethnicity (n = 432, 97.1%), overweight with a BMI ≥ 25 kg/m^2^ (median BMI 27.9 kg/m^2^), regular or occasional alcohol drinkers (n = 326, 73%), current or previous smokers (n = 226, 51%), on cholesterol lowering medication (n = 246, 55.3%) and on blood pressure medication (n = 245, 55.1%). Blood pressure IQRs were close to or the same as elevated blood pressure ranges, i.e. SBP of 120–139 mmHg or DBP of 70–89 mmHg, as defined by the most recent European Society of Cardiology guidelines [45]. These trends were conserved in both sexes, though a higher proportion of males compared to females consumed alcohol more frequently, were current or previous smokers, and took either insulin, blood pressure medication or cholesterol-lowering medication. However, there was a higher proportion of women with a history of cancer compared to men (25.3% vs. 16.7%, *p* < 0.05). Full cohort characteristics are reported in Table 2.

**Table 2:** Demographic, socio-economic, lifestyle, and clinical characteristics of participants in the study sample, stratified by sex

	All n = 445		Males n = 299 (67%)		Females n = 146 (33%)		*p*-value
	Count (%) or Median (Q1-Q3)	Missing (%)	Count (%) or Median (Q1-Q3)	Missing (%)	Count (%) or Median (Q1-Q3)	Missing (%)	
*Demographics*							
Age, years	70.0 (66.0–74.0)	0 (0.0%)	71.0 (66.0–74.0)	0 (0.0%)	70.0 (65.2–73.0)	0 (0.0%)	0.09
Ethnicity	–	0 (0.0%)	–	0 (0.0%)	–	0 (0.0%)	0.16
White	432 (97.1%)	–	289 (96.7%)	–	143 (97.9%)	–	–
Black	1 (0.2%)	–	0	–	1 (0.7%)	–	–
Asian	6 (1.3%)	–	6 (2.0%)	–	0	–	–
Mixed	1 (0.7%)	–	0	–	1 (0.7%)	–	–
Other	5 (1.1%)	–	4 (1.3%)	–	1 (0.7%)	–	–
*Socio-economic background*							
Have college or university degree	66 (14.8%)	0 (0.0%)	45 (15.1%)	0 (0.0%)	21 (14.4%)	0 (0.0%)	0.97
Employment status	–	0 (0.0%)	–	0 (0.0%)	–	0 (0.0%)	0.64
Employed	73 (16.4%)	–	52 (17.4%)	–	21 (14.4%)	–	–
Retired	329 (73.9%)	–	217 (72.6%)	–	112 (76.7%)	–	–
Use private healthcare	72 (16.2%)	0 (0.0%)	48 (16.1%)	0 (0.0%)	24 (16.4%)	0 (0.0%)	1
*Lifestyle characteristics*							
BMI, kg/m^2^	27.9 (24.9–32.0)	15 (3.4%)	28.4 (25.1–31.6)	10 (3.3%)	27.0 (24.4–32.1)	5 (3.4%)	0.09
Waist circumference, cm	95.0 (85.4–105.0)	14 (3.1%)	98.0 (90.0–107.0)	10 (3.3%)	87.0 (78.0–98.0)	4 (2.7%)	< 0.001
Waist-to-hip ratio	0.9 (0.9–1.0)	14 (3.1%)	1.0 (0.9–1.0)	10 (3.3%)	0.8 (0.8–0.9)	4 (2.7%)	< 0.001
Alcohol	–	0 (0.0%)	–	0 (0.0%)	–	0 (0.0%)	< 0.001
Never	30 (6.7%)	–	16 (5.4%)	–	14 (9.6%)	–	–
≤ 3 times a month	89 (20.0%)	–	46 (15.4%)	–	43 (29.5%)	–	–
1–2 times weekly	99 (22.2%)	–	59 (19.7%)	–	40 (27.4%)	–	–
3–4 times weekly	123 (27.6%)	–	96 (32.1%)	–	27 (18.5%)	–	–
Daily	104 (23.4%)	–	82 (27.4%)	–	22 (15.1%)	–	–
Smoking	–	9 (2.0%)	–	5 (1.7%)		4 (2.7%)	0.001
Never	210 (47.2%)	–	124 (41.5%)	–	86 (58.9%)	–	–
Previous	208 (46.7%)	–	158 (52.8%)	–	50 (34.2%)	–	–
Current	18 (4.0%)	–	12 (4.0%)	–	6 (4.1%)	–	–
Unknown	9 (2.0%)	–	5 (1.7%)	–	4 (2.7%)	–	–
*Clinical measurements*							
SBP, mm/Hg	142.0 (130.0–155.1)	69 (15.5%)	142.5 (130.0–157.0)	48 (16.1%)	140.0 (130.5–151.5)	21 (14.4%)	0.18
DBP, mm/Hg	76.0 (70.0–84.0)	69 (15.5%)	76.5 (71.0–84.0)	48 (16.1%)	75.0 (68.0–83.5)	21 (14.4%)	0.17
HDL-C, mmol/L	1.3 (1.1–1.5)	54 (12.1%)	1.2 (1.0–1.4)	3–4 (11.4%)	1.5 (1.2–1.8)	20 (13.7%)	< 0.001
Blood glucose, mmol/L	5.0 (4.7–5.5)	54 (12.1%)	5.0 (4.7–5.5)	34 (11.4%)	5.0 (4.7–5.4)	20 (13.7%)	0.35
Triglycerides, mmol/L	1.6 (1.1–2.3)	23 (5.2%)	1.7 (1.2–2.4)	11 (3.7%)	1.4 (1.0–1.9)	12 (8.2%)	< 0.001
*Medication*							
Cholesterol lowering medication	246 (55.3%)	0 (0.0%)	188 (62.9%)	0 (0.0%)	58 (39.7%)	0 (0.0%)	< 0.001
Blood pressure medication	245 (55.1%)	0 (0.0%)	179 (59.9%)	0 (0.0%)	66 (45.2%)	0 (0.0%)	0.005
Insulin	13 (2.9%)	0 (0.0%)	9 (3.0%)	0 (0.0%)	4 (2.7%)	0 (0.0%)	1
*Medical history*							
Cardiomyopathy	14 (3.1%)	0 (0.0%)	9 (3.0%)	0 (0.0%)	5 (3.4%)	0 (0.0%)	1
Cancer (all types)	87 (19.6%)	0 (0.0%)	50 (16.7%)	0 (0.0%)	37 (25.3%)	0 (0.0%)	0.04
Lung cancer	2 (0.4%)	0 (0.0%)	1 (0.3%)	0 (0.0%)	1 (0.7%)	0 (0.0%)	1
Breast cancer	16 (3.6%)	0 (0.0%)	–	–	16 (11.0%)	0 (0.0%)	–

Statistical tests were used to compare characteristics between male and female subgroups. All continuous variables were compared using the Mann–Whitney U-test aside from waist-to-hip ratio and SBP which were assessed using the independent t-test. Categorical variables were compared using the Chi-square test. The *p*-values reported refer to the individual results of those respective tests. For sex-specific columns, percentage for each count is reported as the percent of that sex subgroup. HFpEF: heart failure with preserved ejection fraction; IQR: inter-quartile range; BMI: body mass index; SBP: systolic blood pressure; DBP: diastolic blood pressure; HDL-C: high-density lipoprotein cholesterol

### Multimorbid cardiometabolic disease patterns differ in men and women with HFpEF

**Fig. 1:**
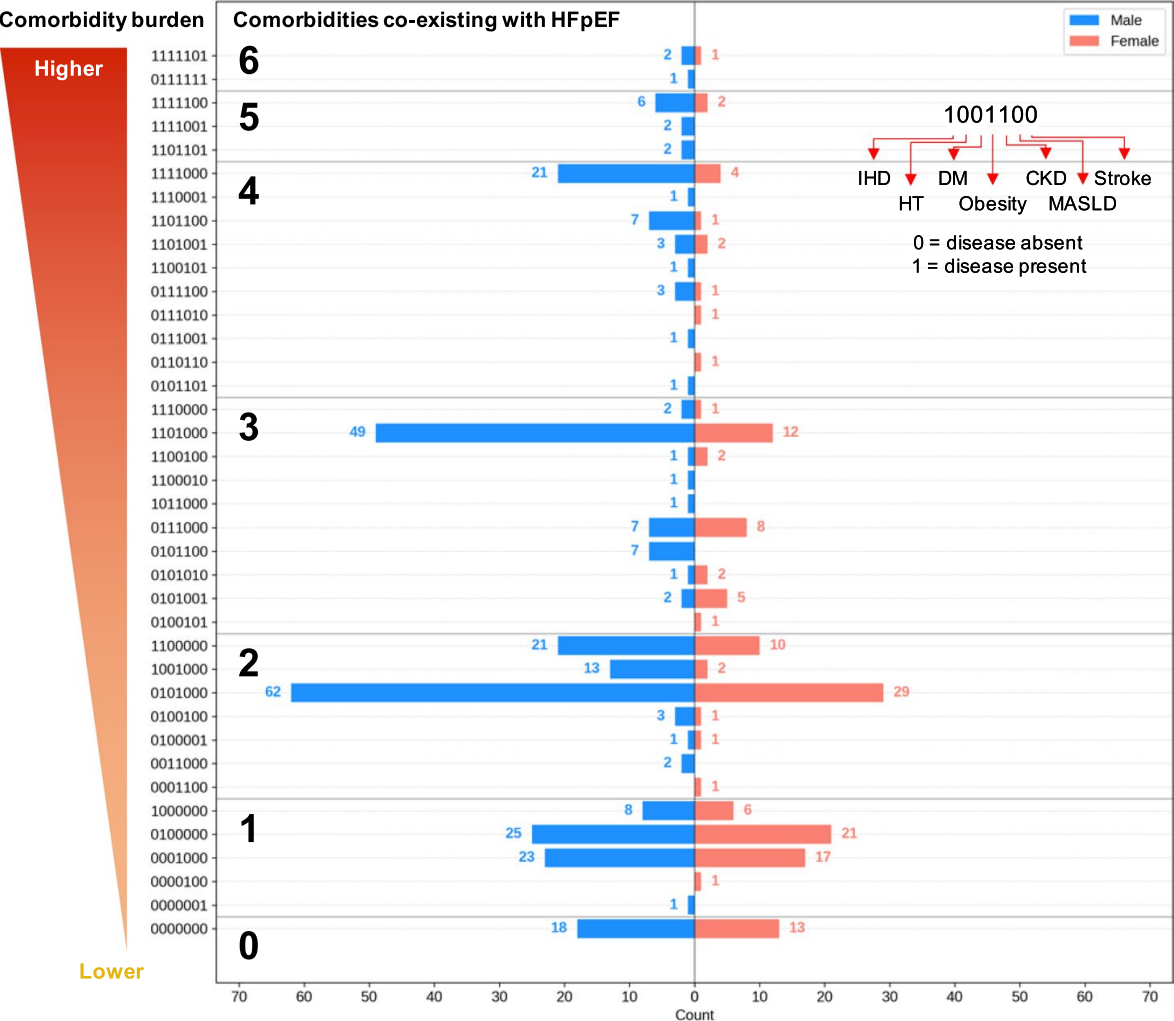
Cardiometabolic comorbidity burden in male (n = 299) and female (n = 146) HFpEF patients. Labels on the y-axis are coded with binary digits indicating the presence (1) or absence (0) of cardiometabolic comorbidities in the following order: ischemic heart disease, hypertension, diabetes, obesity, chronic kidney disease, MASLD, stroke. HFpEF: heart failure with preserved ejection fraction, IHD: ischemic heart disease, HT: hypertension, DM: diabetes mellitus, CKD: chronic kidney disease, MASLD: metabolic dysfunction-associated fatty liver disease.

The sex-specific distribution of cardiometabolic diseases and metabolic syndrome co-existing with HFpEF in our cohort is reported in Table 3, with the exact comorbidity burden in men and women presented in Fig. 1. In our cohort, 281 of 299 (94%) men and 133 of 146 (91%) women were found to have at least one other cardiometabolic disease co-existing with HFpEF, and 224 of 299 (75%) men and 88 of 146 (60%) women had two or more cardiometabolic comorbidities. We also found that the most likely number of comorbidities co-existing with HFpEF was differently distributed between men and women (*p* = 0.05), with the majority of men having 2 (n = 102, 34.1%) or 3 (n = 71, 23.7%) co-morbidities while most women had 1 (n = 45, 30.8%) or 2 (n = 44, 30.1%).

**Table 3:** Cardiometabolic comorbidity burden and metabolic syndrome in the study sample, stratified by sex

	All n = 445	Males n = 299	Females n = 146	
	Count (%)	Count (%)	Count (%)	*p*-value
*MetS components and clinically-defined syndrome*				
Waist circumference > 102 cm (M) or > 89 cm (F)	180 (40.4%)	113 (37.8%)	67 (45.9%)	0.13
BP > 130/85 mmHg	335 (75.3%)	226 (75.6%)	109 (74.7%)	0.92
Triglycerides > 150 mg/dl	197 (44.3%)	148 (49.5%)	49 (33.6%)	0.002
HDL-C < 40 mg/dl (M) or < 50 mg/dl (F)	120 (27.0%)	81 (27.1%)	39 (26.7%)	1
Glucose > 100 mg/dl	86 (19.3%)	63 (21.1%)	23 (15.8%)	0.23
Clinically-defined MetS (≥ 3 components)	146 (32.8%)	103 (34.4%)	43 (29.5%)	0.34
*Cardiometabolic comorbidities*				
Ischemic heart disease	184 (41.3%)	141 (47.2%)	43 (29.5%)	0.001
Hypertension	339 (76.2%)	233 (77.9%)	106 (72.6%)	0.26
Diabetes	68 (15.3%)	49 (16.4%)	19 (13.0%)	0.43
Obesity	304 (68.3%)	216 (72.2%)	88 (60.3%)	0.02
CKD	46 (10.3%)	34 (11.4%)	12 (8.2%)	0.39
MASLD	7 (1.6%)	3 (1.0%)	4 (2.7%)	0.33
Stroke	28 (6.3%)	18 (6.0%)	10 (6.8%)	0.90
Number of additional comorbidities*				0.05
0 (HFpEF only)	31 (7.0%)	18 (6.0%)	13 (8.9%)	**–**
1	102 (22.9%)	57 (19.1%)	45 (30.8%)	**–**
2	146 (32.8%)	102 (34.1%)	44 (30.1%)	**–**
3	102 (22.9%)	71 (23.7%)	31 (21.2%)	**–**
4	48 (10.8%)	38 (12.7%)	10 (6.8%)	**–**
5	12 (2.7%)	10 (3.3%)	2 (1.4%)	**–**
6	4 (0.9%)	3 (1.0%)	1 (0.7%)	**–**

The Chi-square test was used to compare categorical variables between male and female subgroups. The *p*-values reported refer to the individual results of those respective tests. For sex-specific columns, percentage for each count is reported as the percent of that sex subgroup. *Number of additional comorbidities refers to the number of cases with *n* cardiometabolic diseases as defined in main text, additionally to HFpEF, and excluding MetS which we consider separately. HFpEF: heart failure with preserved ejection fraction; MetS: metabolic syndrome

The most common specific comorbidity pattern was hypertension and obesity combined, both in men (n = 62, 21%) and women (n = 29, 20%). Other comorbidity patterns differed in men vs. women (Fig. 1). We found a significant difference in the distribution of obesity and ischemic heart disease cases in men vs. women (n = 141 (47.2%) vs n = 43 (29.5%), *p* = 0.001, and n = 216 (72.2%) vs 88 (60.3%), *p* = 0.02, respectively).

### Latent class analysis reveals three distinct clusters based on cardiometabolic diseases and metabolic syndrome components in HFpEF

No pair-wise associations with a Phi coefficient > 0.4 were observed so we retained all candidate indicator variables for model optimisation (Supplementary Fig. 1). The optimal number of latent classes based on the given indicator variables was found to be 3, based on agreement between AIC and BIC (Supplementary Fig. 3). The results of the LR test were not significant but we have included test results for completeness and transparency (Supplementary Fig. 3).

**Fig. 2:**
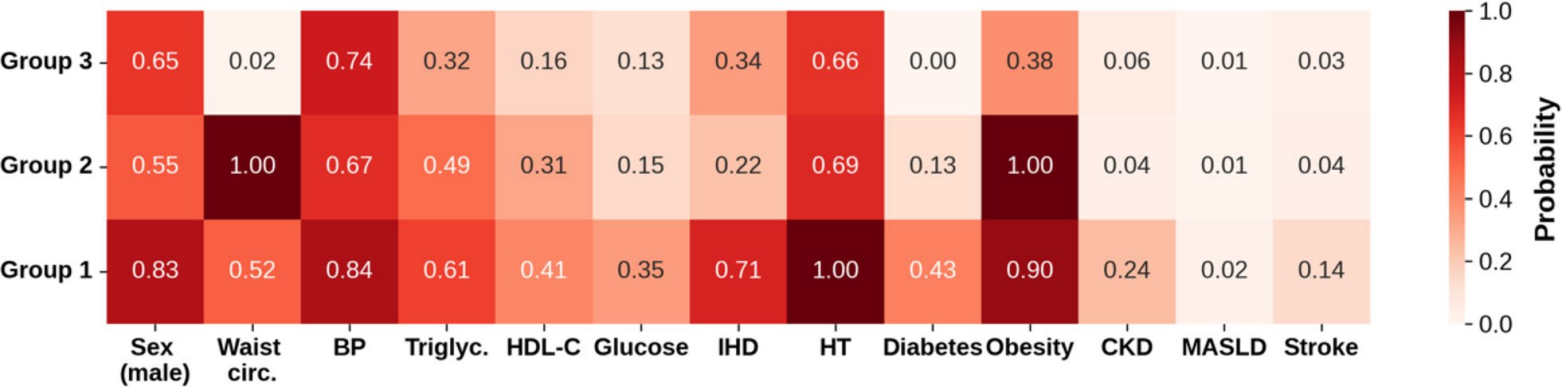
Latent class analysis results: estimated class-conditional probability of each variable being positive in respective classes. By a positive variable response, we refer here to sex being male, individual MetS components being beyond their respective normal clinical thresholds, and individual cardiometabolic diseases being present. Group 1 n = 117, group 2 n = 116, group 3 n = 212. MetS: metabolic syndrome, circ.: circumference, BP: blood pressure, triglyc.: triglycerides, HDL-C: high-density lipoprotein cholesterol, IHD: ischemic heart disease, HT: hypertension, DM: diabetes mellitus, CKD: chronic kidney disease, MASLD: metabolic dysfunction-associated fatty liver disease.

Of the 445 patients in the cohort, 117 (26%) were assigned to Group 1, 116 (26%) to group 2 and 212 (48%) to group 3. The breakdown of class-conditional probability estimates of each variable used to perform LCA is given in Fig. 2. Group 1 was characterised by the highest probability of a patient having a record of singular or co-morbid ischemic heart disease, hypertension, diabetes, CKD and stroke, as well as the highest likelihood of any abnormal metabolic syndrome component, aside from waist circumference. Group 2 was characterised by the highest probability of a patient having obesity and an abnormally high waist circumference. Group 3 was characterised by absolute or relatively low probabilities of patients having any cardiometabolic disease or abnormal metabolic syndrome components, aside from hypertension and high BP which were comparable to Group 2. Group 1 had the highest likelihood of a patient being male (84%). In proportion, there were fewer men in Groups 2 (55%) and 3 (65%) compared to the number of men in the entire cohort (299 of 445 patients, 67%).

We found several significant differences in socio-demographic, lifestyle, and clinical characteristics across the three subgroups (Table 4). Group 1 was found to be the most elderly subgroup, also with the highest median SBP, blood glucose and triglycerides, lowest HDL-C levels, and highest proportion of medicated patients including cholesterol lowering medication, blood pressure medication and insulin. Patients in Group 2 had the highest BMI and waist circumference, which is expected considering the obese profile of the group. Finally, patients in Group 3 had the highest proportion of non-smokers, both previous and current, highest HDL-C and lowest triglyceride levels.

**Table 4:** Latent class analysis results: socio-demographic, lifestyle, and clinical characteristics compared across the different clusters identified

	Group 1 n = 117	Group 2 n = 116	Group 3 n = 212		
	Median (Q1-Q3) or Count (%)	Median (Q1-Q3) or Count (%)	Median (Q1-Q3) or Count (%)	*p*-value (raw)	*p*-value (adjusted)
*Socio-demographics*					
Sex, male	102 (87.2%)	60 (51.7%)	137 (64.6%)	< 0.001	< 0.001
Age, years	71.0 (68.0–75.0)	69.0 (64.0–72.0)	71.0 (66.0–74.0)	0.002	0.183, 0.001, 0.095
Ethnicity	–	–	–	0.58	1.0
White	112 (95.7%)	115 (99.1%)	205 (96.7%)	–	–
Black	1 (0.9%)	0 (0.0%)	0 (0.0%)	–	–
Asian	2 (1.7%)	0 (0.0%)	4 (1.9%)	–	–
Mixed	0 (0.0%)	0 (0.0%)	1 (0.5%)	–	–
Other	2 (1.7%)	1 (0.9%)	2 (0.9%)	–	–
*Lifestyle characteristics*					
BMI, kg/m^2^	30.4 (27.5–33.6)	32.7 (29.6–35.8)	24.9 (23.2–26.9)	< 0.001	< 0.001, 0.001, < 0.001
Waist circumference, cm	101.0 (95.0–110.0)	105.0 (98.8–114.0)	85.0 (80.0–92.0)	< 0.001	< 0.001, 0.028, < 0.001
Waist to hip ratio	1.0 (0.9–1.0)	0.9 (0.9–1.0)	0.9 (0.8–0.9)	< 0.001	< 0.001, 0.113, < 0.001
Alcohol	–	–	–	0.21	0.63
Never	10 (8.5%)	6 (5.2%)	14 (6.6%)	–	–
≤ 3 times a month	19 (16.2%)	29 (25.0%)	41 (19.3%)	–	–
1–2 times weekly	28 (23.9%)	33 (28.4%)	38 (17.9%)	–	–
3–4 times weekly	33 (28.2%)	24 (20.7%)	66 (31.1%)	–	–
Daily	27 (23.1%)	24 (20.7%)	53 (25.0%)	–	–
Smoking	–	–	–	0.02	0.05
Never	45 (39.1%)	55 (47.8%)	110 (53.4%)	–	–
Previous	67 (58.3%)	51 (44.3%)	90 (43.7%)	–	–
Current	3 (2.6%)	9 (7.8%)	6 (2.9%)	–	–
*Clinical measurements*					
SBP, mm/Hg	150.2 (138.6–160.0)	138.8 (125.8–153.6)	139.0 (129.0–152.5)	< 0.001	< 0.001, 0.001, 1.0
DBP, mm/Hg	76.5 (70.5–84.0)	79.5 (70.0–86.6)	75.0 (69.6–82.0)	0.02	1.0, 0.68, 0.05
HDL-C, mmol/L	1.1 (1.0–1.3)	1.3 (1.0–1.5)	1.4 (1.2–1.7)	< 0.001	< 0.001, 0.001, 0.039
Blood glucose, mmol/L	5.4 (4.8–5.9)	4.9 (4.7–5.3)	4.9 (4.6–5.3)	< 0.001	< 0.001, < 0.001, 1.0
Triglycerides, mmol/L	2.0 (1.4–2.5)	1.8 (1.4–2.3)	1.4 (1.0–1.9)	< 0.001	< 0.001, 0.69, < 0.001
*Medication*					
Cholesterol lowering medication	96 (82.1%)	52 (44.8%)	98 (46.2%)	< 0.001	< 0.001
Blood pressure medication	94 (80.3%)	60 (51.7%)	91 (42.9%)	< 0.001	< 0.001
Insulin	12 (10.3%)	1 (0.9%)	0 (0.0%)	< 0.001	< 0.001

Statistical tests were used to compare characteristics across the three groups. Continuous variables were assessed for normality using the Shapiro–Wilk test. Normally distributed variables were compared across the three groups using one-way analysis of variance (ANOVA). Non-normally distributed variables were compared using the Kruksal-Wallis test. Post-hoc pairwise *p*-values were obtained using the Tukey HSD test after ANOVA, or Dunn’s test for the Kruksal-Wallis test, and further adjusted using Bonferroni correction (adjusted *p*-values for continuous comparisons between groups 1 vs. 2, 2 vs. 3, and 1 vs. 3, respectively). Categorical variables were compared using the Chi-square test and also adjusted using Bonferroni correction. Percentage for each count is calculated relative to each respective subgroup. HFpEF: heart failure with preserved ejection fraction; IQR: inter-quartile range; BMI: body mass index; SBP: systolic blood pressure; DBP: diastolic blood pressure; HDL-C: high-density lipoprotein cholesterol

### Left ventricular function across identified HFpEF cardiometabolic phenogroups

**Fig. 3:**
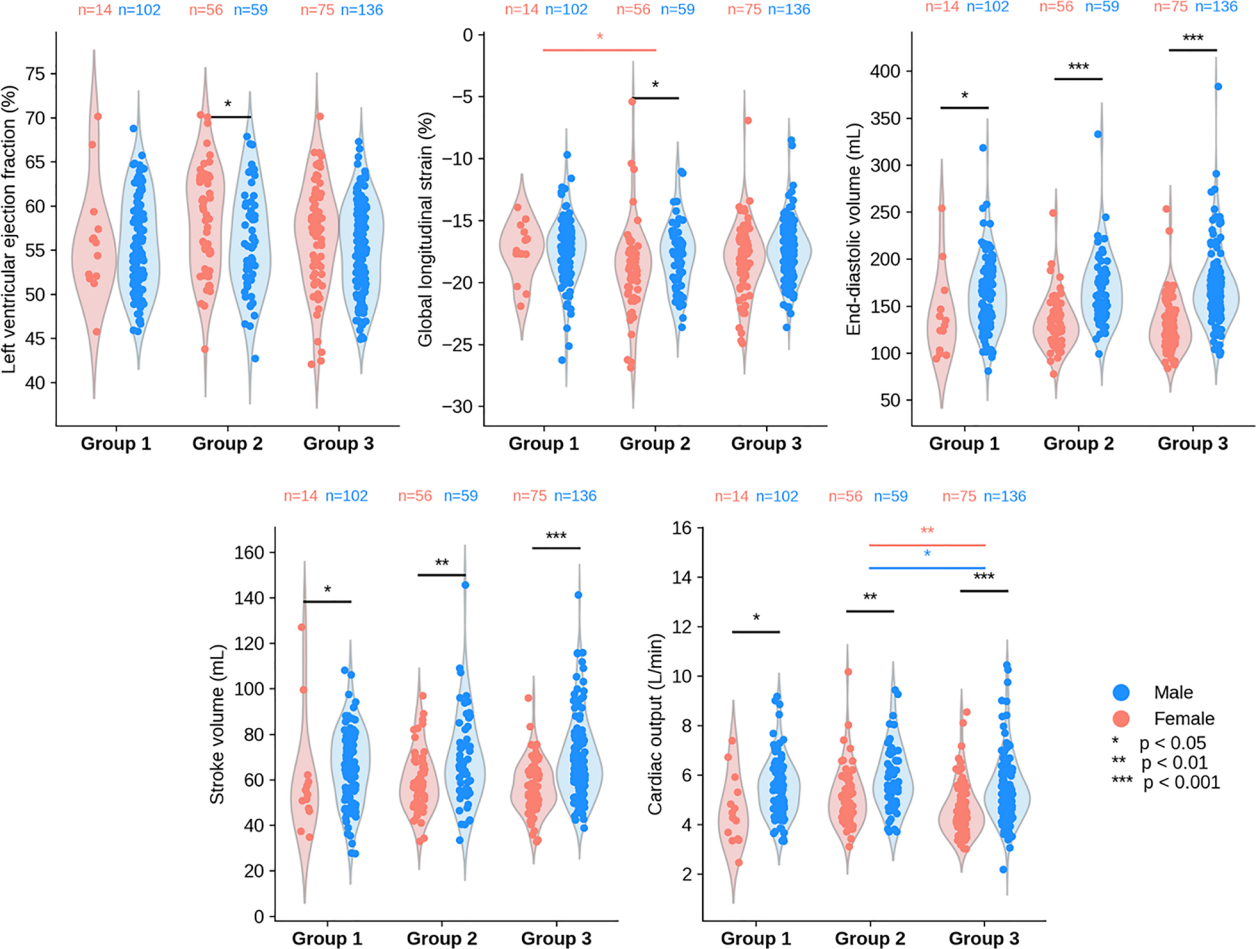
Distribution of CMR image-derived left ventricular function parameters in the HFpEF cardiometabolic phenogroups identified. For normally distributed parameter distributions of sample size n, independent samples t-test was performed only if n > 30 to preserve the validity of the Central Limit Theorem assumption. For non-normal distributions, the Mann Whitney U-test holds regardless of sample size. CMR: cardiac magnetic resonance; HFpEF: heart failure with preserved ejection fraction.

We compared LVEF, global longitudinal strain, LVEDV, LV stroke volume, and cardiac output across the three groups identified by LCA in the HFpEF cohort in the previous stage. Three outlier cases (LVEDV ≥ 500 ml) were removed and 30 cases had one or more missing parameter values, which we imputed. Results of this analysis are visualised in Fig. 3. Results of the same analysis, repeated after excluding cases without a full set of imaging parameters instead of imputation, are available in Supplementary Fig. 4. Both analyses yielded very similar results.

Cardiac output was significantly higher in men and women in Group 2 vs. Group 3 (males: median 5.6 L/min vs. 5.2 L/min, *p* < 0.05; females: 5.1 L/min vs. 4.4 L/min, *p* < 0.01). Absolute global longitudinal strain was significantly lower in women in Group 1 vs. Group 2 (-17.6% vs. -18.5%, *p* < 0.05). There were no statistically significant differences in LVEF, LVEDV or stroke volume across the three groups.

Significant sex-specific differences within each group were observed for LVEDV, stroke volume and cardiac output. These parameters in men were consistently higher than in women, consistent with known underlying sex-dependent anatomical differences in cardiac chamber volumes. Men and women had significantly different distributions of LVEF in Group 2, with higher LVEF in women (59% vs. 56%, *p* < 0.01). Global longitudinal strain differed significantly within Group 2 with higher absolute values observed in women (-18.5% vs. -17.7%, *p* < 0.05), suggesting that contractility in females with HFpEF is similar to males with HFpEF except from Group 2.

Finally, we did not observe any statistically strong relationships between individual metabolic syndrome components i.e. waist circumference, blood pressure, triglycerides, HDL cholesterol and blood glucose, with LVEF, global longitudinal strain, LVEDV, stroke volume, and cardiac output in our cohort (Supplementary Fig. 5).

### Subgroup analysis: prevalent vs incident HFpEF

We compared the cardiometabolic profile of patients with prevalent and incident HFpEF at the time of imaging (Supplementary Table 1). There were very little differences between the two, with the only significant differences found between those two subgroups being a higher proportion of patients in the incident HFpEF subgroup, compared to prevalent HFpEF, with BP levels considered abnormally high in metabolic syndrome terms (n = 227 (79.1%) vs. n = 108 (68.4%)), and more CKD diagnoses (n = 37 (12.9%) vs. n = 9 (5.7%)). These findings suggest that patients with prevalent HFpEF at the time of imaging and incident HFpEF (mean follow-up of 3.3 years), have a similar cardiometabolic burden at the time of imaging. This strongly supports our rationale of considering these jointly for the purposes of this study and its design. However, these results must still be interpreted within the context and limitations of our study and the wider UK Biobank, which we discuss in the following section.

## Discussion

This study provides an in-depth analysis of cardiometabolic HFpEF by examining the cardiometabolic comorbidity burden and metabolic syndrome in a retrospective cohort of elderly men and women with HFpEF, and assessing the relation between different cardiometabolic profiles and cardiac function and hemodynamics. We found that the combination of hypertension and obesity was the most common comorbidity pattern both in men and women with HFpEF, but that the following most common comorbidity patterns in men and women were different. Most men had 2–3 clinical cardiometabolic comorbidities while most women had 1–2, despite a similar profile in terms of abnormal metabolic syndrome components.

Given patients’ cardiometabolic diseases and metabolic syndrome profile, we identified three distinct clusters of HFpEF patients, namely one with the highest likelihood of being male, elderly, and being multimorbid with an abnormal metabolic profile (n = 117); a group with a high prevalence of severe obesity and abnormal waist circumference with the lowest relative proportion of females (n = 116); and a final group with an apparently lower comorbidity burden aside from hypertension, generally “healthier” clinical measurements and the highest proportion of non-smokers (n = 212). Certain characteristics observed in these three clusters overlap with some of the key clinical HFpEF phenotypes that have been proposed previously, namely complex multimorbid HFpEF [36, 38], obese HFpEF [50], and hypertensive HFpEF [51]. Importantly, however, these phenotypical cluster characteristics are not always mutually exclusive, hence the importance of probabilistic grouping and, in more practical terms, a holistic consideration of different clinical characteristics of HFpEF when assessing individual patients.

Women had higher LVEF compared to men in the subgroup with high prevalence of severe obesity. Global longitudinal strain in women was similar to men except in the highly obese subgroup, where men had worse contractility. Cardiac output was significantly higher in the more obese group compared to the least comorbid group, and global longitudinal strain was lower, i.e. contractility was worse, in women in the highly comorbid subgroup compared to the more obese subgroup.

### Challenges in management and treatment of multimorbid HFpEF

Cardiometabolic HFpEF is considered the most prevalent and highest-risk form of HFpEF [21, 3]. Our results highlight the existence of a broad variety of highly multimorbid, abnormal metabolic profiles within the HFpEF population studied. These systemic disturbances are likely to drive a poorer quality of life, symptoms, and higher non-cardiovascular death rates associated with HFpEF [52, 6]. SGLT2 inhibitors are the only existing therapy with proven benefit in HFpEF patients; a benefit that is due to lower HF hospitalisation rates, but with no significant reduction of cardiovascular mortality nor clinically meaningful improvements in quality-of-life scores [53, 51, 54]. Our findings strengthen the need to pursue a personalised, phenotype-specific approach to optimally manage HFpEF. Furthermore, patients with obesity and HFpEF have been shown to have an exacerbated symptom burden, be more prone to exercise intolerance, and have higher rates of hospitalisation for HF [55, 56, 57]. Identifying and addressing modifiable risk factors such as BMI and obesity may help to control disease progression and improve adverse outcomes in HFpEF. Our study provides a further clinical characterisation of the cardiometabolic HFpEF phenotype, supporting our understanding of the variety of patient profiles that exist within cardiometabolic HFpEF including obesity, and thus contributing to a better identification of different patient subgroups eligible for optimal therapeutic strategies.

### Sex-specific differences in HFpEF rates and cardiometabolic profiles

A study by Fry et al. presented evidence of a “healthy volunteer” bias among the UK Biobank study population, with the prevalence of self-reported conditions being lower than that of the general UK population as reported in national health surveys [58]. One particular finding suggests that this bias is even more pronounced for cases of cardiovascular disease reported in women compared to men: only 7.4% of women aged 45–64 in the UK Biobank were reported as having cardiovascular disease, as opposed to 25.5% in the general population, while rates for men in the same age range were 16.1% in the UK Biobank vs 29.4% in the general population. The “healthy volunteer” bias explained by Fry et al., exacerbated in sex-specific reports of cardiovascular disease, could possibly explain the unexpected sex-specific distribution of HFpEF cases identified in our UK Biobank cohort, despite our subset of participants with imaging data, regardless of HF status, consisting of 52% women. However, we still found that women with HFpEF were more likely to have a lower comorbidity burden compared to men, which aligns with previously published studies [59, 60].

### HFpEF, diabetes and chronic kidney disease

The pathophysiological pathways involved in the cardio-renal-metabolic axis all contribute to HFpEF and the adverse progression of connected systemic diseases, including diabetes and CKD [36, 51]. A recent meta-analysis of large-scale HFmrEF (HF with mildly reduced EF) and HFpEF clinical trials highlighted the high prevalence of comorbid diabetes and CKD in those cohorts, with rates as high as 35–40% in four major trials. We found comparatively low rates of diabetes (n = 68, 15.3%) and CKD (n = 46, 10.3%). One plausible explanation for this discrepancy is that HFpEF is commonly underdiagnosed in patients with type 2 diabetes and CKD in the general population [61, 62, 63]. Indeed, challenges in HFpEF diagnosis may be responsible for unrecognised HFpEF in UK Biobank participants with diabetes or CKD. The lack of complete linkage of the UK Biobank to primary care records (as of early 2025) could also be responsible for missing cases of diabetes and CKD, as both diseases are more likely to be identified in primary care settings [40].

### Clinical relevance of global longitudinal strain and cardiac output in HFpEF

Impaired global longitudinal strain (GLS), i.e. a lower absolute value, is common in HFpEF. It is associated with reduced contractility and diastolic dysfunction, and is a strong predictor of adverse events and disease progression [32, 33]. Studies have shown that obese patients and those with higher BMI have impaired GLS [64], and that metabolic syndrome is also associated with impaired GLS, with central obesity being the most strongly associated component [65]. We found that women in the most highly comorbid subgroup had a significantly lower GLS absolute value compared to those in the subgroup with highest rates of obesity and abnormal waist circumference. Women in the obese subgroup had a higher absolute GLS value compared to men in the same subgroup, which aligns with previous studies showing that GLS tends to be higher in women than men [66, 67] and that male sex is an independent predictor of GLS decline [68]. This expected sex-specific difference, however, was not observed in subgroup 1 with high comorbidity burden nor in subgroup 3 with a high likelihood of hypertension. These heterogenous results highlight the importance of considering sex and existing comorbidities beyond obesity as important factors in the interpretation of GLS in the context of HFpEF.

A reduced cardiac output, notably during exercise, is one of the hallmarks of HFpEF [69, 70]. At rest, cardiac output tends to be normal in HFpEF, but variations may occur due to underlying or comorbid conditions [71]. We found that cardiac output measured non-invasively at rest was lower in Group 3 (apparently “healthier” but hypertensive) compared to Group 2 (obese and abnormal waist circumference). This finding may be reflective of high-output HF, a form of HF that typically results from the body’s increased demand for blood resulting from increased metabolic activity and/or excessive vasodilatation, which are both common in obesity [72]. A previous study also showed that increasing severity of obesity in HFpEF patients was associated with a higher cardiac output at rest, which further supports our findings [73].

### Novelty compared to existing HFpEF phenotyping studies

Unlike previous HFpEF studies, our clustering approach is based exclusively on sex and cardiometabolic disturbances, the main pathophysiological drivers of HFpEF. Previous studies approach clustering from a much wider angle, typically based a broader set of variables, aside from a few exceptions– one study clustered HFpEF patients based on > 300 blood-based biomarkers [31], while another clustered based on non-cardiac conditions, frailty and nutrition indicators in the elderly [24]. We considered CMR imaging-derived parameters beyond the LVEF as study outcomes, to compare cardiac health in the different phenogroups identified. A couple of other studies also considered imaging parameters as outcomes, however neither included global longitudinal strain despite its clinical relevance in HFpEF [21, 31]. In contrast, other studies considered these parameters as clustering variables rather than outcomes [22, 74, 25, 26, 27, 20, 28], or did not include them in their analyses [23, 24, 29, 30]. Finally, the use of LCA and probabilistic grouping in our study provides advantages over k-means or hierarchical clustering approaches previously used [25, 26, 30, 31].

### Strengths and limitations

A key strength of this study comes from the richness and quality of UK Biobank data, which was recorded following a robustly validated and systematic data recording protocol, helping to minimise data collection bias. The multi-centre nature of the UK Biobank allows for better geographical coverage and diversity of the study population at a national level. The depth of data available enabled an extensive characterisation of each case in our study sample.

However, the constraints imposed by our research question limited our cohort to include only participants with CMR data, a record of HF, and an EF ≥ 50%. Due to the timing of our study in the lifespan of the UK Biobank, this led to a relatively small HFpEF cohort, however we hope this may pave the way for larger and possibly longitudinal, comparative studies of HFpEF in the UK Biobank in the future as more cases arise. Incomplete linkage to primary care data in the UK Biobank is another limitation which we discussed in context earlier. The small cohort size may weaken the reliability and generalisability of statistical analyses, especially for subgroup analyses. In addition, HFpEF develops very gradually and is challenging to diagnose, meaning that the date at which it was first recorded clinically may not always capture the condition at consistent stages of disease progression across different patients. We acknowledge the newly emerging definition of obesity as a clinical illness linked to organ dysfunction due to excess adiposity [75]. However, this definition has not been widely adopted in medical practice yet, and ascertaining “clinical obesity” is complex and beyond the scope of this paper, therefore we decided to define obesity using traditional BMI-based criteria. Data on exercise intolerance could provide more information on the difference between subgroups, however the UK Biobank did not collect imaging data under exercise conditions. This would be important to consider in the design of future biobanks or cohort studies of HFpEF. Finally, causal inferences cannot be made given the design of the study, and the study did not include an external validation cohort. Due to these limitations, further phenotyping studies are warranted to validate these results, especially when translating findings to HFpEF populations of different sex, socio-economic background and ethnicity, to address any biases that may exist due to the demographics of the UK Biobank.

The challenges of obtaining both large cohorts and “deep” i.e. multi-modal data for highly robust HFpEF phenotyping studies are widely acknowledged. To tackle this, the UK HFpEF Collaborative Group and the US National Heart, Lung and Blood Institute (NHLBI), for example, have set up specific consortia to aggregate multiple existing HFpEF data sources and collect new data across a range of clinical modalities in order to enable larger-scale, more effective HFpEF phenotyping research and trials to take place [76, 77, 78].

## Conclusion

Our study provides focused insights into the distribution of cardiometabolic multimorbidity burden and metabolic syndrome profiles of men and women with HFpEF in an elderly cohort from the UK Biobank. Latent class analysis based on patients’ cardiometabolic profile yielded three distinct, clinically relevant phenogroups, namely: an older, more male and multimorbid subgroup; a subgroup with a high prevalence of severe obesity with the highest relative proportion of females; and a generally healthier yet hypertensive subgroup with a less severe cardiometabolic profile. We found significant differences in body shape and mass measurements, lipid biomarkers and medication across the different groups identified. Differential analysis of imaging-derived parameters of left ventricular function suggested a higher cardiac output in the most obese subgroup, as well as higher global longitudinal strain in women compared to men in this subgroup. Coupled with current challenges and limitations of HFpEF treatment, our work aligns with the vision of personalised medicine by providing further evidence supporting phenotype-specific classification and subsequent disease management routes, especially regarding the control of modifiable yet highly prevalent risk factors such as hypertension and obesity. Further studies in larger cohorts are warranted to validate these findings, especially in women and populations of different ethnicities.

## Supplementary Information

Supplementary Material 1.

## Data Availability

The data underlying this study were provided by the UK Biobank upon application. Access to UK Biobank data for research purposes can be obtained upon application (https://www.ukbiobank.ac.uk/). This study was conducted under UK Biobank Application Number 116292.
