# Supplementary material for: Sex-specific cardiometabolic multimorbidity, metabolic syndrome and left ventricular function in heart failure with preserved ejection fraction in the UK Biobank
Source: Cardiovasc Diabetol. 2025 Jun 4;24:238. doi: 10.1186/s12933-025-02788-4 (PMC12139127; doi:10.1186/s12933-025-02788-4)
Supplement: Supplementary file 1 — Supplementary Material 1. [file 12933_2025_2788_MOESM1_ESM.docx]

**Supplementary Figure 1.** Variable selection for latent class analysis. Pairwise Phi coefficients computed to assess the association between candidate indicator variables to include in the latent class analysis models. BP: blood pressure, HDL: high-density lipoprotein, IHD: ischemic heart disease, HT: hypertension, CKD: chronic kidney disease, MASLD: metabolic dysfunction-associated fatty liver disease.


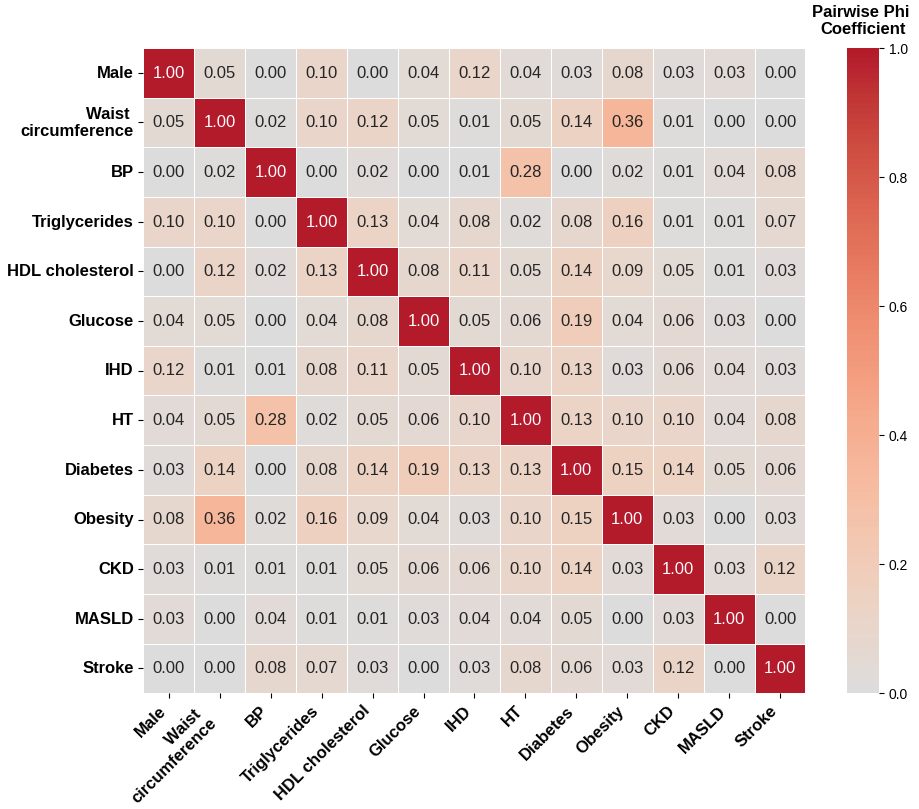


**Supplementary Figure 2.** Latent class analysis model optimisation: Akaike Information Criterion (AIC) and Bayesian Information Criterion (BIC) computed for candidate models with an increasing number of latent classes, ranging from 1 to 7. The optimal model was found to have n=3 classes, which minimises BIC and is further supported by AIC(n=3) < AIC(n=2).


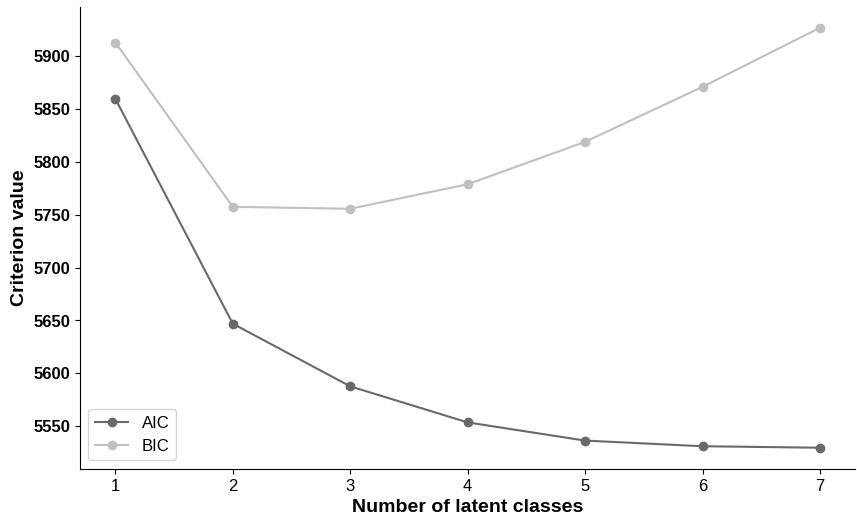


**Supplementary Figure 3.** Latent class analysis model optimisation. Likelihood Ratio (LR) test comparing model fit for candidate models with an increasing number of latent classes, ranging from 2 to 7. The LR test did not provide further significant results to consider additionally to the Akaike Information Criterion (AIC) and Bayesian Information Criterion (BIC).

**
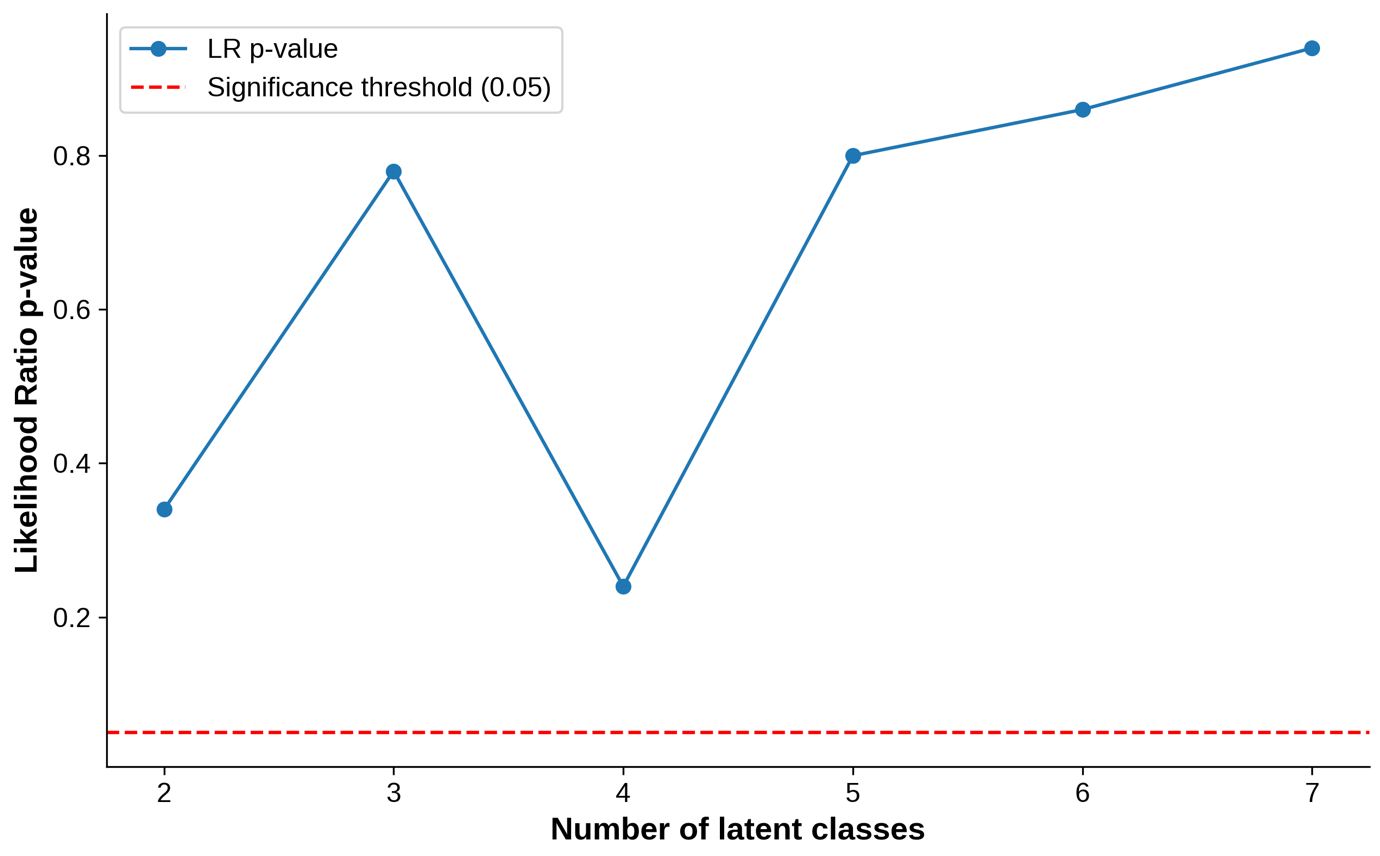
**

**Supplementary Figure 4.** Distribution of CMR image-derived left ventricular function parameters in the HFpEF cardiometabolic phenogroups identified, excluding cases without a full set of imaging parameters. For normally distributed parameter distributions of sample size n, independent samples t-test was performed only if n>30 to preserve the validity of the Central Limit Theorem assumption. For non-normal distributions, the Mann Whitney U-test holds regardless of sample size. CMR: cardiac magnetic resonance; HFpEF: heart failure with preserved ejection fraction.


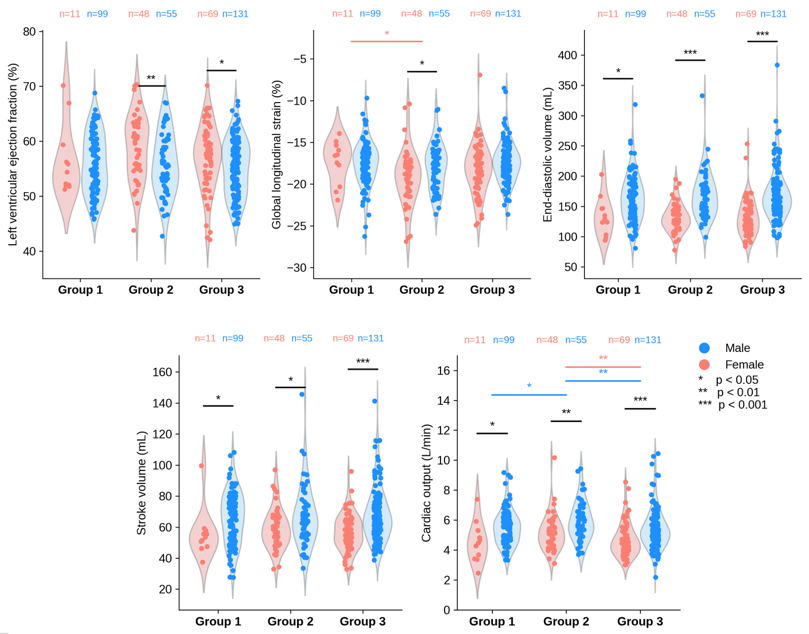


**Supplementary Figure 5.** Left ventricular function parameters according to individual metabolic components in males and females with HFpEF. HFpEF: heart failure with preserved ejection fraction; LVEF: left ventricular ejection fraction; HDL: high-density lipoprotein.


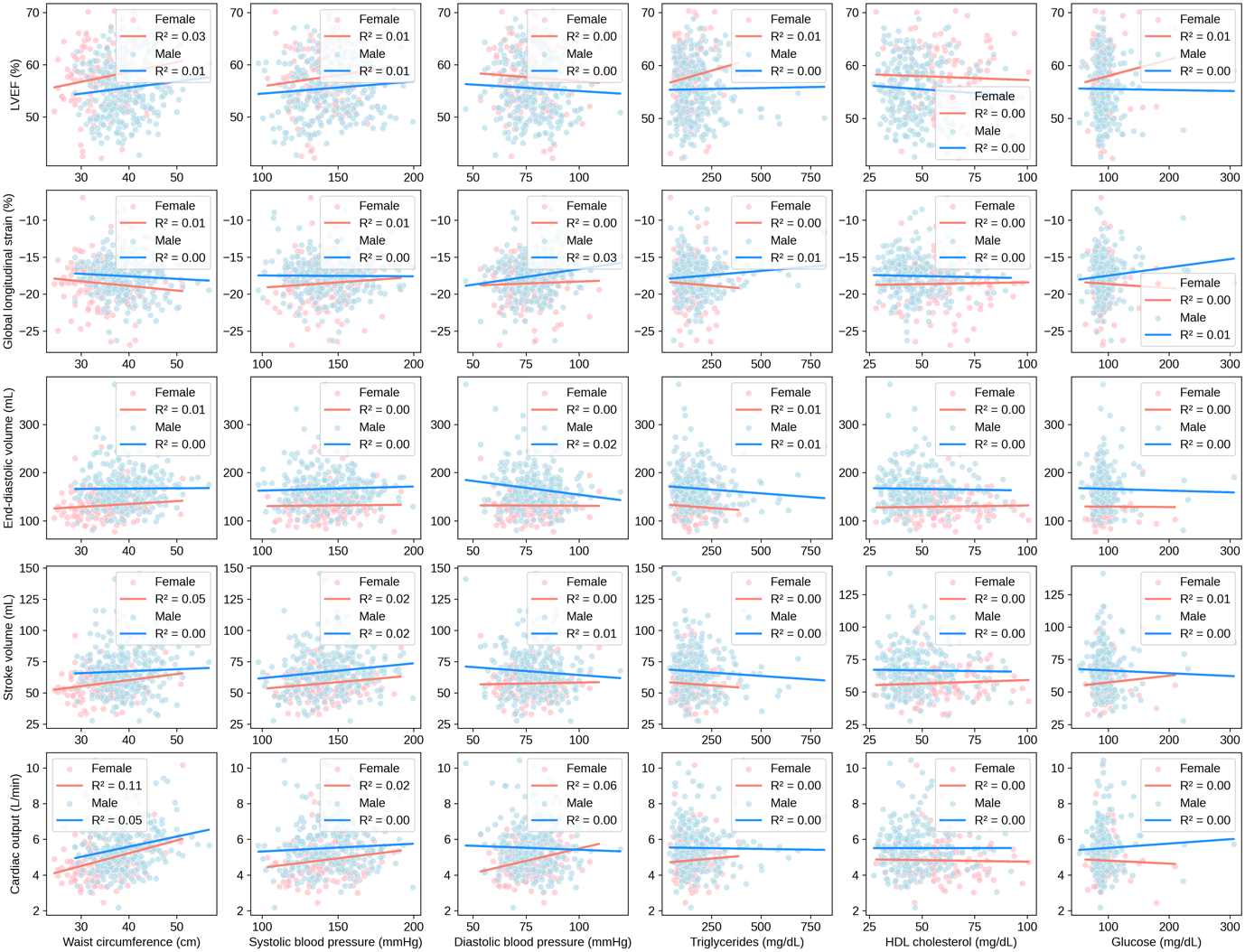


**Supplementary Table 1.** Cardiometabolic comorbidity burden and metabolic syndrome in our study cohort, stratified by HFpEF status (incident vs prevalent). The Chi-square test was used to compare categorical variables between incident and prevalent subgroups. The p-values reported refer to the individual results of those respective tests. Percentage for each count is reported as the percent of that subgroup. *Number of additional comorbidities refers to the number of cases with *n* cardiometabolic diseases as defined in main text, additionally to HFpEF, and excluding MetS which we consider separately. M: male; F: female; HFpEF: heart failure with preserved ejection fraction; MetS: metabolic syndrome.

|  | **Incident HFpEF**  **n = 287** | **Prevalent HFpEF**  **n = 158** |  |
| --- | --- | --- | --- |
|  | **Count (%)** | **Count (%)** | **p-value** |
| **MetS components and**  **clinically-defined syndrome** |  |  |  |
| Waist circumference >102 cm (M) or >89 cm (F) | 107 (37.3%) | 73 (46.2%) | 0.083 |
| BP >130/85 mmHg | 227 (79.1%) | 108 (68.4%) | 0.016 |
| Triglyceride >150 mg/dl | 127 (44.3%) | 70 (44.3%) | 1 |
| HDL-C <40 mg/dl (M) or <50 mg/dl | 71 (24.7%) | 49 (31.0%) | 0.188 |
| Glucose >100mg/dl | 57 (19.9%) | 29 (18.4%) | 0.795 |
| Clinically-defined MetS (≥ 3 components) | 87 (30.3%) | 59 (37.3%) | 0.16 |
| **Cardiometabolic comorbidities** |  |  |  |
| Ischemic heart disease | 109 (38.0%) | 75 (47.5%) | 0.065 |
| Hypertension | 226 (78.7%) | 113 (71.5%) | 0.11 |
| Diabetes | 51 (17.8%) | 17 (10.8%) | 0.067 |
| Obesity | 197 (68.6%) | 107 (67.7%) | 0.926 |
| CKD | 37 (12.9%) | 9 (5.7%) | 0.026 |
| MASLD | 5 (1.7%) | 2 (1.3%) | 1 |
| Stroke | 23 (8.0%) | 5 (3.2%) | 0.07 |
| Number of additional comorbidities* |  |  | 0.57 |
| *0 (HFpEF only)* | 19 (6.6%) | 12 (7.6%) |  |
| *1* | 64 (22.3%) | 38 (24.1%) |  |
| *2* | 91 (31.7%) | 55 (34.8%) |  |
| *3* | 67 (23.3%) | 35 (22.2%) |  |
| *4* | 32 (11.1%) | 16 (10.1%) |  |
| *5* | 11 (3.8%) | 1 (0.6%) |  |
| *6* | 3 (1.0%) | 1 (0.6%) |  |
